# STRIKE-GOLDD 4.0: user-friendly, efficient analysis of structural identifiability and observability

**DOI:** 10.1093/bioinformatics/btac748

**Published:** 2022-11-18

**Authors:** Sandra Díaz-Seoane, Xabier Rey Barreiro, Alejandro F Villaverde

**Affiliations:** Department of Systems and Control Engineering, Universidade de Vigo, Galicia, 36310 Vigo, Spain; Department of Systems and Control Engineering, Universidade de Vigo, Galicia, 36310 Vigo, Spain; Department of Systems and Control Engineering, Universidade de Vigo, Galicia, 36310 Vigo, Spain; CITMAga, Galicia, 15782 Santiago de Compostela, Spain

## Abstract

**Motivation:**

STRIKE-GOLDD is a toolbox that analyses the structural identifiability and observability of possibly non-linear, non-rational ODE models that may have known and unknown inputs. Its broad applicability comes at the expense of a lower computational efficiency than other tools.

**Results:**

STRIKE-GOLDD 4.0 includes a new algorithm, ProbObsTest, specifically designed for the analysis of rational models. ProbObsTest is significantly faster than the previously available FISPO algorithm when applied to computationally expensive models. Providing both algorithms in the same toolbox allows combining generality and computational efficiency. STRIKE-GOLDD 4.0 is implemented as a Matlab toolbox with a user-friendly graphical interface.

**Availability and implementation:**

STRIKE-GOLDD 4.0 is a free and open-source tool available under a GPLv3 license. It can be downloaded from GitHub at https://github.com/afvillaverde/strike-goldd.

**Supplementary information:**

Supplementary data are available at Bioinformatics online.

## 1 Introduction

A model is structurally identifiable (respectively, observable) if it is theoretically possible to determine its parameters (respectively, state variables) by observing its output (Wieland *et al.*, 2021). Many software tools have been developed for the analysis of these properties (Bellu *et al.*, 2007; Dong *et al.*, 2021; Hong *et al.*, 2019; Karlsson *et al.*, 2012; Ligon *et al.*, 2018; Meshkat *et al.*, 2014; Sedoglavic, 2002; Shi and Chatzis, 2022; Villaverde *et al.*, 2016); their performance has been assessed by Rey Barreiro and Villaverde (2022). Structural identifiability and observability (SIO) can be tested locally or globally. Most local tools are based on an algorithm by Sedoglavic (2002), which is computationally fast but can only be applied to rational models. In contrast, the STRIKE-GOLDD toolbox implements an algorithm called FISPO that is usually less efficient, but allows analysing non-rational models. While this versatility is the main strength of STRIKE-GOLDD, it comes at the expense of higher computation times for rational models than other toolboxes tailored to that problem class. To address this issue we present STRIKE-GOLDD 4.0, which introduces two new features. First, it implements ProbObsTest, an extension of the algorithm by Sedoglavic (2002) for the analysis of rational models, for which it can achieve considerable speed-ups over FISPO. As a second main feature, STRIKE-GOLDD 4.0 is implemented as a Matlab toolbox with a user-friendly graphical interface.

## 2 Methods and implementation

**Fig. 1.**
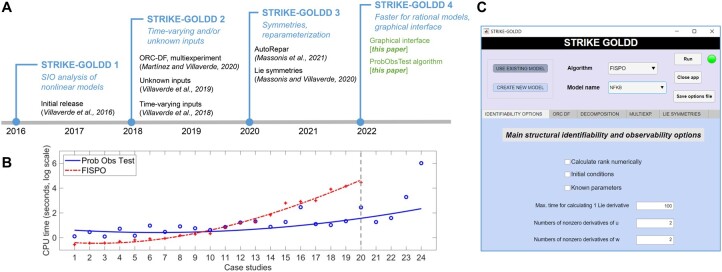
(**A**) Timeline of the main releases of STRIKE-GOLDD. Each version provides an integrated solution with significantly new features with respect to the previous one, which are summarized in italics below each main version along with their publication. (**B**) Computation times, in seconds, of the new algorithm (ProbObsTest) and the previously existing one (FISPO), for 24 case studies of increasing computational complexity. Results were obtained on a computer with an Intel Core i7-11700KF processor of 3.60 GHz, 16 GB RAM, running Matlab R2021b on Windows. Note that the vertical axis is shown in logarithmic scale. The models and their characteristics are detailed in the Supplementary Material. Circles represent individual computation times, lines are interpolations. The vertical grey line indicates that FISPO cannot analyse the last four models, due to computational limitations. (**C**) Graphical interface of the Matlab app STRIKE-GOLDD 4.0.

STRIKE-GOLDD follows a differential geometry approach to the SIO analysis of systems of nonlinear ordinary differential equations (ODE). Figure 1A shows a timeline of its developments. STRIKE-GOLDD 4.0 provides three algorithms for SIO analysis. The most generally applicable, FISPO (Villaverde *et al.*, 2019), can analyse non-rational models and, by extending the observability rank condition (ORC; Hermann and Krener, 1977), models with unknown inputs. Models that are affine in the inputs can also be analysed with a second algorithm, ORC-DF (Maes *et al.*, 2019).

STRIKE-GOLDD 4.0 includes a third algorithm, ProbObsTest, which is based on the algorithm presented by Sedoglavic (2002) for rational models. It accelerates the ORC test by avoiding the symbolic computation of Lie derivatives. ProbObsTest presents two main developments with respect to the algorithm on which it is based. First, it can analyse models with unknown polynomial inputs; second, it can automatically transform certain non-rational models—such as those with logarithmic and trigonometric functions, or non-integer exponents—into rational models. Details are provided in the Supplementary Material. We note that a previous extension of Sedoglavic’s algorithm for models with unknown inputs was presented by Shi and Chatzis (2022) and is available at https://eng.ox.ac.uk/non-lineardynamics/resources/. Unlike ProbObsTest, it does not assume polynomial inputs.

Figure 1B shows a comparison of the computation times of ProbObsTest and FISPO for the analysis of 24 models of increasing complexity. For Models 1–10, which can be analysed in a few seconds or minutes, ProbObsTest is slightly slower than FISPO (note the logarithmic scale). However, for the more computationally expensive models (11–24) ProbObsTest is faster, and the difference is larger as the models become more complex. Indeed, ProbObsTest, unlike FISPO, is able to analyse Models 21–24; notably, the latter has 117 parameters. Details of the case studies are provided in the Supplementary Material, alongside a report of the advantages of using the new algorithm in the toolbox. Comparisons to other methods can be found in Rey Barreiro and Villaverde (2022).

A second new feature of STRIKE-GOLDD 4.0 is its implementation as a Matlab toolbox with a graphical interface, a screenshot of which is shown in Figure 1C. This new interface coexists with the previously existing way of executing the toolbox, which is by running a Matlab script; in this case, one must indicate the settings by editing an options file. A user manual is provided in the documentation folder of STRIKE-GOLDD 4.0.

## 3 Conclusion

STRIKE-GOLDD is arguably the most generally applicable toolbox for local SIO analysis, being able to handle ODE models that may be non-rational and not affine in the inputs, and which may admit unknown external inputs. Previously, this generality came at the expense of high computation times for larger rational models. STRIKE-GOLDD 4.0 provides a method for a faster analysis of such models, along with a more generally applicable one. The coexistence of both algorithms, as well as the other features of the toolbox (searching for Lie symmetries and suggesting reparameterizations), makes it a convenient multi-purpose tool for the analysis and reformulation of systems biology models. Its usability is enhanced by its implementation as a Matlab app with a user-friendly graphical interface.

## Supplementary Material

btac748_Supplementary_Data
