# Supplementary material for: STRIKE-GOLDD 4.0: user-friendly, efficient analysis of structural identifiability and observability
Source: Bioinformatics. 2022 Nov 18;39(1):btac748. doi: 10.1093/bioinformatics/btac748 (PMC9805590; doi:10.1093/bioinformatics/btac748)
Supplement: btac748_Supplementary_Data [file btac748_supplementary_data.pdf]

SUPPLEMENTARY INFORMATION
# of the paper
"STRIKE-GOLDD 4.0: user-friendly, efficient analysis of structural identifiability and observability"

Sandra Díaz-Seoane[1], Xabier Rey Barreiro[1], Alejandro F. Villaverde[1,2]
[1] Universidade de Vigo, Department of Systems Engineering & Control, 36310 Vigo, Galicia, Spain
[2] CITMAga, 15782 Santiago de Compostela, Galicia, Spain

1 Theory and methods

1.1 Background on Structural Identifiability and Observability (SIO)

Structural identifiability and observability are two key concepts in system identification and dynamic modelling. To give their formal definitions we consider models described by ordinary differential equations with general form:

$$M : \begin{cases} \dot{x} = & f\left(u(t), x(t), \theta\right), \\ y = & g\left(u(t), x(t), \theta\right), \end{cases} \tag{1}$$

where f and g are analytic functions (therefore infinitely differentiable); $x(t) \in \mathbb{R}^{n_x}$ is the state variables vector; $u(t) \in \mathbb{R}^{n_u}$, the known inputs vector; $y(t) \in \mathbb{R}^{n_y}$, the outputs vector; $\theta \in \mathbb{R}^{n_\theta}$, the parameters vector. The input vector, $u(t)$, is assumed to consist of infinitely differentiable functions.

A parameter θ_i of M (1) is *structurally locally identifiable* (s.l.i.) if a neighbourhood $\mathcal{N}(\theta^*)$ exists such that, for any $\hat{\theta} \in \mathcal{N}(\theta^*)$, $y(t, \theta^*) = y(t, \hat{\theta})$ holds if and only if $\theta_i^* = \hat{\theta}_i$, for almost any parameter vector $\theta^* \in \mathbb{R}^{n_\theta}$. We say that a parameter is structurally unidentifiable (s.u.) if this relationship does not hold in any $\mathcal{N}(\theta^*)$. If all the parameters of a model are s.l.i., the model is s.l.i. too. Accordingly, if at least one the parameters is s.u., the model is s.u.

A s.l.i. parameter can be determined from knowledge of the output $y(t)$ and input $u(t)$ of the model. Likewise, a state $x_i(\tau)$ is said to be *observable* if it can be determined from the output $y(t)$ and any known inputs $u(t)$ of the model in the interval $t_0 \leq \tau \leq t \leq t_f$, for a finite t_f. Otherwise, it is unobservable. A model is observable if all its states are observable, and unobservable if at least one of them is unobservable.

1.2 SIO analysis with the Observability Rank Condition

Let us begin with the analysis of observability. The available knowledge for inferring the internal state x of model M consists of the output y and its derivatives. Following a differential geometry approach [3, 19] we construct a matrix $\mathcal{O}(x)$ that represents a map between the model output y and its derivatives $\dot{y}, \ddot{y}, ...$, on the one hand, and its state x on the other. We can then evaluate the observability by calculating the rank of $\mathcal{O}(x)$. If it has full rank, then the model is observable. If $rank(\mathcal{O}(x_0)) = n_x$, M is observable around x_0.

With time varying inputs, the output derivatives $\dot{y}, \ddot{y}, ...$ are the so-called "extended" Lie derivatives. The extended Lie derivative [8] of g with respect to f is defined by:

$$\mathcal{L}_f g(x) = \frac{\partial g(x)}{\partial x} f(x, u) + \sum_{j=0}^{j=\infty} \frac{\partial g(x)}{\partial u^{(j)}} u^{(j+1)}$$

and the high order derivatives are recursively calculated:

$$\mathcal{L}_f^i g(x) = \frac{\partial L_f^{i-1} g(x)}{\partial x} f(x, u) + \sum_{j=0}^{j=\infty} \frac{\partial L_f^{i-1} g(x)}{\partial u^{(j)}} u^{(j+1)}$$

The ith Lie derivative may contain input derivatives only up to order $(i-1)$ [24]. We can then truncate the infinite summation at $j = i - 1$ and rewrite the extended Lie derivative:

$$\mathcal{L}_f g(x) = \frac{\partial g(x)}{\partial x} f(x,u) + \sum_{j=0}^{j=i-1} \frac{\partial g(x)}{\partial u^{(j)}} u^{(j+1)}$$

We summarise the way of computing the observability matrix in the following way:

$$\mathcal{O}(x(t)) = \begin{pmatrix} \frac{\partial}{\partial x} y(t) \\ \frac{\partial}{\partial x} \dot{y}(t) \\ \vdots \\ \frac{\partial}{\partial x} y^{(n_x-1)}(t) \end{pmatrix} = \begin{pmatrix} \frac{\partial}{\partial x} g(x) \\ \frac{\partial}{\partial x} \left(\mathcal{L}_f g(x)\right) \\ \vdots \\ \frac{\partial}{\partial x} \left(\mathcal{L}_f^{n_x-1} g(x)\right) \end{pmatrix}. \tag{2}$$

For structural identifiability, parameters can be considered as constant state variables [17]. Assessing the observability of these states is equivalent to assessing the structural identifiability of the parameters. To this end we construct an augmented state vector $\tilde{x} = [x(t); \theta]$ with dimension $n_{\tilde{x}} = n_x + n_\theta$ and then we have that $\dot{\tilde{x}} = [f(\tilde{x}(t), u(t));\ 0]$. For a new model with these changes in the variables and equations we construct now an observability-identifiability matrix, $\mathcal{O}_I(\tilde{x}(t))$, in the same way as $\mathcal{O}(x(t))$. Then, if system M given by (1) satisfies $rank(\mathcal{O}_I(\tilde{x}_0)) = n_{\tilde{x}} = n_x + n_\theta$, with $\tilde{x}_0$ a point in the augmented state space, the model is observable and identifiable around $\tilde{x}_0$.

1.3 SIO analysis with the FISPO algorithm in STRIKE-GOLDD

In [21] we see that full rank of $\mathcal{O}_I$ might be achieved with less than $n_{\tilde{x}} - 1$ Lie derivatives. We also have a minimum number of Lie derivatives, n_d, for which the matrix may be full rank. In STRIKE-GOLDD, the $\mathcal{O}_I$ is recursively calculated. Once the n_d Lie derivative is computed, the rank is calculated after adding each new derivative allowing early termination of the procedure. If the full rank is achieved, the OIC is fulfilled; if the rank stops increasing, there is at least one unobservable state or unidentifiable parameter. In the latter case we can determine which parameter or state is unidentifiable or unobservable, respectively. Each column of $\mathcal{O}_I$ corresponds to the partial derivative with respect to a parameter or state. Removing each of the columns and recalculating the rank allows us to know which of the variables is unidentifiable or unobservable. If the rank does not change when we remove the ith column, then the ith variable is unidentifiable or unobservable.

Some models have unmeasured inputs. This is the case of disturbances or time-varying parameters, for example. To analyse them we define a new model that includes them as additional variables:

$$M_w : \begin{cases} \dot{x} = & f\left(u(t), w(t), x(t), \theta\right), \\ y = & g\left(u(t), w(t), x(t), \theta\right), \end{cases} \tag{3}$$

where $w(t)$ refers to the unknown inputs. Then, we define a property analogous to observability for these variables. An unknown input $w_i(\tau)$ is *reconstructible* if it can be determined from $y(t)$ and $u(t)$ in $t_0 \le \tau \le t \le t_f$, for a finite t_f. A model is reconstructible if all its unknown inputs are reconstructible (or "input observable").

The property that encompasses observability, structural identifiability, and reconstructibility is called FISPO (full input, state, and parameter observability) [24]. Let $\tilde{x}(t) = [x(t), \theta, w(t)]$ be the vector of unknown model quantities (i.e. states, parameters and inputs), with $z(t) \in \mathbb{R}^{n_x+n_\theta+n_w}$, and let us denote each element of $\tilde{x}(t)$ at time τ as $z_i(\tau)$. We say that the model M (1) has the FISPO property if every $\tilde{x}_i(\tau)$ can be determined from the output $y(t)$ and any known inputs $u(t)$ of the model in the interval $t_0 \le \tau \le t \le t_f$, for a finite t_f. Thus, M is FISPO if, for every $\tilde{x}_i(\tau)$, for almost any vector $\tilde{x}^*(\tau)$ there is a neighbourhood $\mathcal{N}(\tilde{x}^*(\tau))$ in which the following holds:

$$\hat{\tilde{x}}(\tau) \in \mathcal{N}(\tilde{x}^*(\tau)) \quad and \quad y(t, \hat{\tilde{x}}(t)) = y(t, \tilde{x}^*(\tau))) \qquad \Rightarrow \qquad \hat{\tilde{x}}_i(\tau) = \tilde{x}_i^*(\tau).$$

For assessing reconstructibility, we consider a new augmented state vector $\bar{x} = [x(t); \theta; w(t)]$ and consequently new state dynamics $\dot{\bar{x}} = [f(\bar{x}(t), u(t)); 0; \dot{w}(t)]$. Now, the ith Lie derivative may

contain derivatives up to $w^{(i)}$ so, we need to include them in the augmented state vector:

$$\bar{x} = \begin{bmatrix} x(t) \\ \theta \\ w(t) \\ \dot{w}(t) \\ \vdots \\ w^{(i)}(t) \end{bmatrix} \tag{4}$$

thus the state dynamics are:

$$\dot{\bar{x}} = \begin{bmatrix} f(\bar{x}(t),u(t)) \\ 0 \\ \dot{w}(t) \\ \dot{w}(t) \\ \vdots \\ w^{(i+1)}(t) \end{bmatrix}. \tag{5}$$

where $\bar{x} \in \mathbb{R}^{n_{\bar{x}}}, n_{\bar{x}} = n_x + n_\theta + n_w(i+1)$.

Let M_w be a model of the form (3). M_w is FISPO if the new matrix $\mathcal{O}_I$ computed with the new augmented state vector $\bar{x}(t)$ (4) and its corresponding equations $\dot{\bar{x}}(t)$ (5) is such that $rank(\mathcal{O}_I(\bar{x}, u)) = n_{\bar{x}}$. If the matrix is not full rank when it is not computationally feasible or convenient to keep calculating Lie derivatives, the result is inconclusive. To deal with this, we may set to zero the derivatives of $w(t)$ of order higher than a given one (i). We will then have that $w^{(j)} = 0, \quad \forall j \geq i$. Even though this assumption restricts the type of inputs that can be analysed, in [24] (page 5, Subsection 2.3) it was suggested that perhaps the results might apply to generic inputs under certain circumstances.

1.4 SIO analysis with a probabilistic algorithm to test local algebraic observability in polynomial time: *ObservabilityTest*

Sedoglavic presented an algorithm [15] related with the differential algebra approach [5], with the goal of computing the set of observable variables of a model in polynomial time. When this technique determines that a variable is observable, the result is guaranteed to be correct. If it classifies it as unobservable, the result is correct with high probability. This approach is applicable to nonlinear rational dynamical systems without unknown inputs. Its definition of algebraic observability is built on the existence of algebraic relations between the state variables and the successive derivatives of the inputs and the outputs. If there is an algebraic relation that allows finitely many trajectories of the state variables that are solutions of the vector field and yield the same specified input-output behavior, the state variables are said to be locally observable [15]. A Maple implementation of this method, called *ObservabilityTest*, is available at https://github.com/sedoglavic/ObservabilityTest/.

Let us now introduce some notation [15] to formalize the previous definition. We use capital letters to denote the initial conditions of a function and its derivatives, i.e., $u^{(r)}(0) = U^{(r)}$ and $y^{(r)}(0) = Y^{(r)}$ for $r \geq 0$ and then $U = (U^{(0)}, U^{(1)}, ...)$, $Y = (Y^{(0)}, Y^{(1)}, ...)$. We denote the field adjoining the indeterminates $U_i^{(0)}, U_i^{(1)}, ...$ for $i = 1, ..., n_u$ and $Y_j^{(0)}, Y_j^{(1)}, ...$ for $j = 1, ..., n_y$ to $\mathbb{R}$ as $\mathbb{R}\langle U, Y\rangle$.

We say that x_i, $i \in \{1, ..., n_x\}$ is locally algebraically observable if x_i is algebraic over the field $\mathbb{R}\langle U, Y\rangle$. The system M (1) is locally algebraically observable if the field extension $\mathbb{R}\langle U, Y\rangle \hookrightarrow \mathbb{R}\langle U, Y\rangle(x)$ is algebraic. The number of non-observable state-variables which should be assumed known, in order to obtain an observable system, can be calculated as the transcendence degree of $\mathbb{R}\langle U, Y\rangle \hookrightarrow \mathbb{R}\langle U, Y\rangle(x)$. In [15] the transcendence degree is also calculated with the rank of $\mathcal{O}(x(t))$ (2). Thus, if it is a full rank matrix, the transcendence degree is zero and the system is algebraically observable. If it is not full rank, then at least one of the variables is not identifiable and further analysis would be needed to determine which one it is.

While the way of analysing the properties is the same as in the FISPO algorithm, the procedure to compute the matrix and its rank is rather different. In this case, a variational system derived from M (1) is used to directly compute the Jacobian matrix $\mathcal{O}$ [15]; with x, θ, and u specialized

on some given values. Let us denote by $\Phi(x,\theta,u,t)$ the formal power series in t with coefficients in $\mathbb{R}\langle u\rangle(x,\theta)$ solution of $\dot{\Phi}=f(\Phi,\theta,u)$ with initial condition $\Phi(x,\theta,u,0)=x$. Then:

$$\Phi(x,\theta,u,t)=x+\sum_{j\in\mathbb{N}^*}\mathcal{L}^j f(x,\theta,u)\frac{t^j}{j!}.$$

Besides, using Φ, we define the formal power series in t with coefficients in $\mathbb{R}\langle u\rangle(x,\theta)$ as:

$$y(x,\theta,u,t)=g(\Phi(x,\theta,u,t),\theta,u,t)=g(x,\theta,u)+\sum_{j\in\mathbb{N}^*}\mathcal{L}^j g(x,\theta,u)\frac{t^j}{j!}$$

Based on this we have:

$$\begin{aligned}\mathcal{O}_{alg}=\frac{\partial(y^{(i)})_{0\le i\le n_{\tilde{x}}}}{\partial(x,\theta)}&=\text{coeffs}\left(\frac{\partial g}{\partial x}\frac{\partial\Phi}{\partial x},\frac{\partial g}{\partial x}\frac{\partial\Phi}{\partial\theta}+\frac{\partial g}{\partial\theta}\right)\\&=\text{coeffs}\left(\nabla y\left(\Phi,\frac{\partial\Phi}{\partial x},\frac{\partial\Phi}{\partial\theta}\right),j^j,j=0,...,n_{\tilde{x}}\right).\end{aligned}\tag{6}$$

where $\nabla y(\Phi,\Gamma,\Lambda,\theta,u)=\left(\frac{\partial g}{\partial x}\Gamma,\frac{\partial g}{\partial x}\Lambda+\frac{\partial g}{\partial x}\right)(\Phi,\Gamma,\Lambda,\theta,u)$. Consequently, we have to compute the $n_{\tilde{x}}$ first terms of the power series expansion of Φ, $\Gamma=\frac{\partial\Phi}{\partial x}$ and $\Lambda=\frac{\partial\Phi}{\partial\theta}$.

Since $P(\dot{x},x,\theta,u)=0$, the numerators of the rational relations $\dot{x}-f(x,\theta,u)=0$ and ∇P:

$$\begin{cases}P(\dot{x},x,\theta,u),\\\frac{\partial P}{\partial\dot{x}}(x,\theta,u)\dot{\Gamma}+\frac{\partial P}{\partial x}((\dot{x},x,\theta,u)\Gamma,\\\frac{\partial P}{\partial\dot{x}}(x,\theta,u)\dot{\Lambda}+\frac{\partial P}{\partial x}((\dot{x},x,\theta,u)\Lambda+\frac{\partial P}{\partial\theta}((\dot{x},x,\theta,u),\end{cases}$$

the power series Φ,Γ and Λ are solutions of the system of ordinary differential equations $\nabla P=0$ with the associated initial conditions $\Gamma(x,\theta,u,0)=Id_{n_x\times n_x}$ and $\Lambda(x,\theta,u,0)=0_{n_x\times n_\theta}$.

Next, we specialize the parameters on some random integer θ^* and the inputs on the power series u^*, which are truncated at order $n_{\tilde{x}}+1$ with random integer coefficients. Then, we solve the associated system $\nabla P=0$ for some integer initial conditions x_0, and we compute with ∇y the specialization of $\mathcal{O}_{alg}$.

The Newton operator used in the algorithm is based on the resolution of the following system of linear ordinary differential equations:

$$\frac{\partial P}{\partial\dot{x}}\dot{E}_{j+1}+\frac{\partial P}{\partial x}E_{j+1}+\nabla P=0\bmod t^{2j+1},\tag{7}$$

with $E_{j+1}=(\Phi-\Phi_j,\Gamma-\Gamma_j,\Lambda-\Lambda_j)\bmod t^{2j+1}$, correction term, with Φ_j, Γ_j and Λ_j are approximations of Φ, Γ and Λ, respectively. The system is solved using (Φ_{j+1}, Γ_{j+1}, Λ_{j+1}) = (Φ_j, Γ_j, Λ_j) + E_{j+1} and the initial conditions $\Phi_0\in\mathbb{Z}^{n_x}$, $\Gamma_0=Id_{n_x\times n_x}$ and $\Lambda_0=0_{n_x\times n_\theta}$. The resolution of the linear ordinary differential system relies on the method of integrating factors. We take the homogeneous system

$$\frac{\partial P}{\partial\dot{x}}(\Phi_j,\theta^*,u^*)\dot{\Omega}_j+\frac{\partial P}{\partial x}(\dot{\Phi}_j,\Phi_j,\theta^*,u^*)\Omega_j+\nabla P=0\bmod t^{2j+1},$$

where Ω_j denotes a $n_x\times n_x$ unknown matrix whose coefficients are truncated series. This homogeneous system is then solved by means of a procedure called "Homogeneous Resolution" [15] based on matricial resolution and, in a similar way, the expression in (7) can be computed with a given precision by a procedure called "Constants Variation" [15]. Algorithm 1 summarizes the procedure.

2 New developments in STRIKE-GOLDD 4.0

2.1 A new algorithm for SIO analysis in STRIKE-GOLDD: *ProbObsTest*

STRIKE-GOLDD 4.0 includes a Matlab implementation of an extended version of Sedoglavic's *ObservabilityTest* algorithm. The new method is called *ProbObsTest*. It is introduced with the aim of complementing the FISPO algorithm, achieving computational acceleration with respect

Preprocesing *Construct a straight-line program encoding the variational system* ∇P *and the expressions used during its integration.*
Specialization *Specialization of the parameters,* θ^**, and the inputs,* u^*
Power Series Solution *Computation of the power series solution of* ∇P *at order* $n_{\tilde{x}} + 1$ *with a specialised value for the states*
Jacobian computation *Evaluation of* ∇y *on the series* Φ_j*,* Γ_j *and* Λ_j *where* $j = ln_2(n_{\tilde{x}} + 1)$*, giving the coefficients of the Jacobian matrix*
Rank computation *Calculation of the matrix rank and transcendence degree*
if *transcendence degree = 0* **then**
| System is algebraically observable
else
| Determine which variable or variables are not observable.
end

Algorithm 1: Probabilistic algorithm to test local algebraic observability in polynomial time

to it. In the FISPO algorithm the Lie derivatives are recursively calculated. When their number is relatively small they can be computed in a feasible amount of time. However, for each new Lie derivative that is needed the computational complexity increases, and calculations quickly become impracticable when the number of unknown variables grows. To avoid the need for such calculations, in our implementation of *ProbObsTest* we use the variational system described by Sedoglavic, substituting all the symbolic variables with random numerical values. After this, we get a polynomial matrix that we vectorize, exploiting MATLAB capabilities, in order to obtain an even more efficient implementation. Thus we perform the computations with numerical matrices instead of symbolic expressions. This yields a new code, whose computational complexity increases at a significantly slower rate than FISPO for complex models (as will be demonstrated in Section 2.4). The remainder of this section describes the new developments included in *ProbObsTest* with respect to the *ObservabilityTest* algorithm originally presented in [15].

2.2 Extending the algorithm to admit unknown polynomial inputs

Sedoglavic's *ObservabilityTest* algorithm cannot analyse models with unknown inputs. An extension of Sedoglavic's algorithm with this purpose has recently been presented in [16]. Similarly to the FISPO algorithm, *ProbObsTest* can handle unknown polynomial inputs by treating them as states. To this end the state is augmented as in (4) and the state function f as in (5). We can include as many input derivatives as additional states as the number of non-zero derivatives specified as options; the last element of vector f will always be a zero in the presence of unknown polynomial inputs. This method cannot handle an infinite number of non-zero derivatives, since the order of the computations grows with the number of derivatives. If the user indicates an infinite number of derivatives in the options, the toolbox automatically lowers it to a relatively small number of non-zero derivatives, warning the user of the change and advising her/him to increase the number if needed, or using the FISPO algorithm to consider the infinite case.

2.3 Automatic model reformulation: obtaining a rational model

In principle, *ObservabilityTest* can only be applied to rational models. In *ProbObsTest* we have included a procedure to replace non-rational expressions appearing in the model equations with their Taylor expansions. This allows applying the algorithm to non-rational models for which such an expansion is possible; while it is not feasible to do the Taylor expansion for all non-rational functions, this solution covers a significant amount of models. The toolbox automatically checks whether it is possible to perform the transformation. If it is, the non-rational expression is replaced by the Taylor expansion truncated at the Matlab default order (six), approximating the expressions with fifth-degree polynomials.. If it is not, the algorithm cannot be applied and an error is issued. When this change is possible, it increases the number of variables to be replaced by random values in the system equations. This may increase the computation time, specially if a large truncation order is set. However, this growth is not a function of the number of unknown variables of the model (which would be problematic, since it could compromise its application to large models)..

The polynomial numerators of rational terms are obtained with the function $rational2poly-nomial$, which we implemented as an adaptation of the *dagnormal* procedure in *ObservabilityTest*. Both functions obtain the numerator and denominator of a rational expression. However, we realised that *ObservabilityTest* has problems when non-integer exponents appear in the equations:

such terms are non-rational, but the algorithm does not detect it. To fix this issue we automatically approximate such values with the closest integer, which allows applying the procedure while obtaining correct results in the general case.

A note of caution. Care should be taken when performing this type of reformulation, since it may change the SIO properties of the model. When that happens, the results of the SIO analysis are valid for the reformulated model, but not necessarily for the original one. The following example illustrates this point. Consider a model with states x_1 and x_2, with the following state equations:

$$\dot{x}_1 = 1,$$

$$\dot{x}_2 = x_2,$$

$$y = x_2 + e^{x_1}$$

We have that $x_1 = t + x_1(0)$ and $x_2 = x_2(0)e^t$. Replacing the values of x_1 and x_2 in the output equation we obtain $y = (x_2(0) + e^{x_1(0)})e^t$. For every value of $x_1(0)$ one can find a value of $x_2(0)$ that produces the same y, so x_1 and x_2 are not locally observable. However, if we transform the model to a polynomial form by replacing e^{x_1} with $p(x_1)$ (where p is a fixed polynomial) in the expression of y, then both x_1 and x_2 become locally observable. Thus, the transformation does not preserve the non-observability of the original model. An alternative approach to rationalise some equations is by adding extra equations and variables, as seen in the supplementary data of [6] (example A.2) and also in [4, 11] . However, this procedure has not been automated yet. In [11] it was demonstrated that, even for this type of exact rationalizations, the unidentifiability of the transformed model does not ensure the unidentifiability for the original one. In contrast, the identifiability of the transformed model entails that the original one is also identifiable. This problem is also seen in example 3 of [4].

2.4 Implementation: a Matlab toolbox with graphical interface

The *ProbObsTest* algorithm has been included in STRIKE-GOLDD 4.0, which has been implemented as a Matlab toolbox. A screenshot of its graphical interface is shown in Fig. 1.C of the main text. The interface allows choosing the algorithm from a drop-down menu and specifying its settings; likewise, it is possible to select one of the models already existing in the folder from a drop-down menu, or creating a new model from scratch in a new window. This new interface coexists with the previously existing way of executing the toolbox, which is by running a Matlab script; in this case one must indicate the settings by editing an options file. Further details can be found in the user manual included in the documentation folder of STRIKE-GOLDD 4.0.

3 Results

3.1 Comparing FISPO and *ProbObsTest*

To determine the computational improvement achieved by *ProbObsTest* with respect to FISPO, we have compared their performance by applying them to a set of 22 problems, which are listed in Table 1. We considered a number of variations of some of them, so as to assess the effect of varying the number of unknown input derivatives on the performance of the algorithms. The CPU times of both algorithms for the set of 22 models are shown in Figure 1.B of the main text; complementary to this, we show in figure 1 a graphic comparing the performance of the two algorithms with respect to the number of unmeasured variables. In the remainder of this section we describe each of the models and discuss the results of the two aforementioned algorithms while also comparing them, when possible, with *ObservabilityTest*.

C2M model: The model corresponds to a two compartment system appearing in [22, 24]. The model consists of:

- 2 states, $x = (x_1; x_2)$,
- 4 parameters, $\theta = (k_{1e}; k_{12}; k_{21}; b)$,
- 1 input, u,

Table 1: List of benchmark models and their main features.

Model	Ref.	States	Param.	Known inputs	Unknown inputs (nonzero derivatives)	Outputs
1: C2M 1	[22, 24]	2	2	0	1(0)	1
2: HIV 3	[24, 13]	3	5	1	0	2
3: C2M 2	[22, 24]	2	4	1	0	1
4: 2DOF 1	[10]	4	3	1	1(0)	2
5: C2M 3	[22, 24]	2	3	0	1(0)	1
6: 2DOF 2	[10]	4	3	1	1(2)	2
7: C2M 4	[22, 24]	2	2	0	1(3)	1
8: 2DOF 3	[10]	4	3	0	2(0)	2
9: PK 1	[14]	4	10	1	0	2
10: C2M 5	[22, 24]	2	3	0	1(3)	1
11: PK 2	[14]	4	10	0	1(0)	2
12: PK 3	[14]	4	10	0	1(3)	2
13: 2DOF 4	[10]	4	3	0	2(3)	2
14: SIRS	[2]	5	10	0	0	2
15: MARKOV 1	[22]	2	10	1	0	1
16: NF-κB 1	[9, 21]	15	13	1	0	6
17: βIG 1	[7]	3	5	1	0	1
18: HIV 5	[25]	5	10	0	0	2
19: βIG 2	[7, 20]	3	5	0	1(0)	1
20: NF-κB 2	[9, 21]	15	29	1	0	6
21: βIG 3	[7]	3	5	0	1(3)	1
22: MARKOV 2	[22]	2	10	1	0	1
23: JAK-STAT	[1]	25	27	5	0	14
24: CHO	[23]	32	117	0	0	13

- 1 output, x_1,

and it is governed by the following state equations:

$$f(x, \theta, u, w) = \begin{pmatrix} -(k_{1e} + k_{12}) \cdot x_1 + k_{21} \cdot x_2 + b \cdot u \\ k_{12} \cdot x_1 - k_{21} \cdot x_2 \end{pmatrix}.$$

We start considering the input as known, obtaining a FISPO result from both algorithms. Then, we assume the input as unknown and parameter b as known. Finally, we also assume k_{1e} as known. *ObservabilityTest* can not be applied when there is an unknown input. For the other two methods we also distinguish between constant input and with three non-zero derivatives.

For the known input case the analyses with *ObservabilityTest*, FISPO and *ProbObsTest* are done in 0,047 seconds, 0,38 seconds and 1,25 seconds, respectively.

With an unknown constant input (i.e. considered as an additional parameter), considering b and k_{1e} known, the analyses take 0,28 seconds with FISPO and 1,29 seconds with *ProbObsTest*. With an unknown input with 3 non-zero derivatives, it takes 0,87 seconds with FISPO and 2,96 seconds with *ProbObsTest*.

Finally, with k_{1e} unknown, the analysis with an unknown constant input takes 0,67 seconds with FISPO and 1,40 seconds with *ProbObsTest*. With 3 non-zero derivatives it takes 2,14 seconds with FISPO and 4,19 seconds with *ProbObsTest*.

HIV model: This dynamic system represents a HIV virus infection. We consider two different types of this model. The first one, presented and analysed in [24, 13], consists of:

- 3 states, $x = (T_u; T_I; V)$,
- 5 parameters, $\theta = (\lambda; \rho; N; \delta; c)$,
- 1 know input, η,
- 2 outputs, $g(x, \theta, w) = (V; T_I + T_u)$,

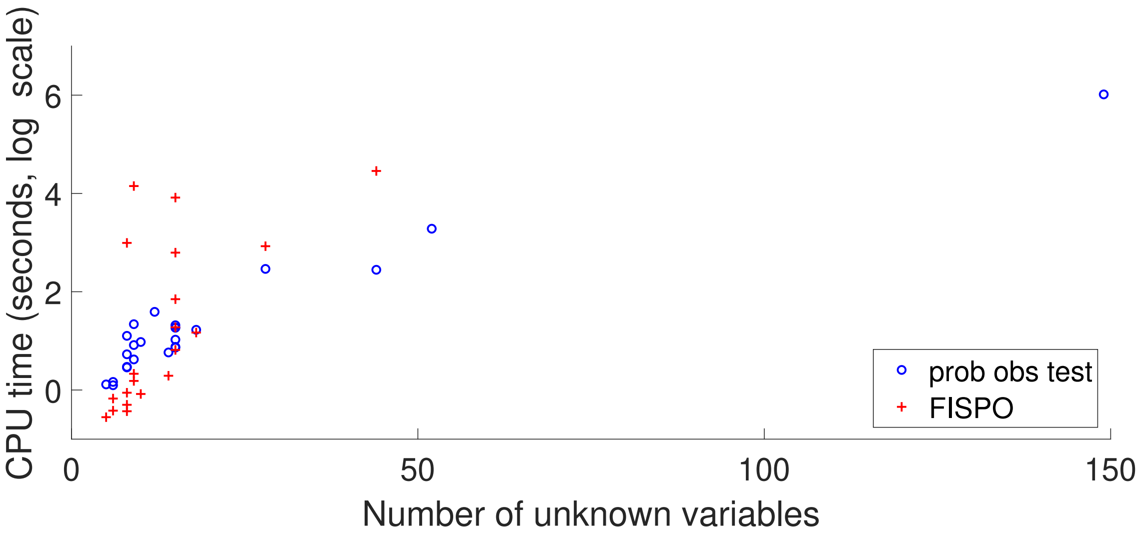

Figure 1: Comparison of times of FISPO and *ProbObsTest* with respect to the number of unmeasured variables.

and it is governed by the following state equations:

$$f(x,\theta,u,w) = \begin{pmatrix} \lambda - \rho \cdot T_u - \eta \cdot T_u \cdot V \\ \eta \cdot T_u \cdot V - \delta \cdot T_I \\ N \cdot \delta \cdot T_I - c \cdot V \end{pmatrix}.$$

The three algorithms yield a FISPO result. *ProbObsTest* takes 2,88 seconds, STRIKE-GOLDD implementation 0,37 and *ObservabilityTest* 0,093.

The other model is introduced in [25]. It has:

- 5 states, $x = (xx; y; v; ww; z)$,
- 10 parameters, $\theta = (\beta; \lambda; a; b; c; d; hh; k; q; uu)$,
- 0 inputs,
- 2 outputs, $g(x,\theta,w) = (ww; z)$,

and it is governed by the following state equations:

$$f(x,\theta,u,w) = \begin{pmatrix} \lambda - (d \cdot xx) - (\beta \cdot xx \cdot v) \\ (\beta \cdot xx \cdot v) - (a \cdot y) \\ (k \cdot y) - (uu \cdot v) \\ (c \cdot z \cdot y \cdot ww) - (c \cdot q \cdot y \cdot ww) - (b \cdot ww) \\ (c \cdot q \cdot y \cdot ww) - (hh \cdot z) \end{pmatrix}.$$

The results obtained are the same for the three algorithms: 3 non observable states and 4 non identifiable parameters. FISPO takes 8237,7 seconds, *ProbObsTest* 10,57 and *ObservabilityTest* 0,203.

2DOF model: This model is introduced and studied in [10]. It is an affine-in-the-inputs model that characterizes the behavior of a mechanical system. The model consists of:

- 4 states, $x = (x_1; x_2; \dot{x_1}; \dot{x_2})$,
- 3 parameters, $\theta = (k_1; \delta k_1; m_2)$,
- 1 known input, $u = F_1$,
- 1 unknown input, $w = F_2$,
- 2 outputs,

and it is governed by the following state equations:

$$f(x, \theta, u, w) = \begin{pmatrix} \dot{x_1} \\ \dot{x_2} \\ (-(k_1 + \delta \cdot k_1 \cdot x_1)x_1 + k_2 \cdot (x_2 - x_1) - c_1 \cdot \dot{x_1} + c_2 \cdot (\dot{x_2} - \dot{x_1}) + F_1)/m_1 \\ (k_2 \cdot (x_1 - x_2) + c_2 \cdot (\dot{x_1} - \dot{x_2}) + F_2)/m_2 \end{pmatrix},$$

and the output equations:

$$g(x, \theta, w) = \begin{pmatrix} x_1 \\ (k_2 \cdot (x_1 - x_2) + c_2 \cdot (\dot{x_1} - \dot{x_2}) + F_2)/m_2 \end{pmatrix}.$$

As we have an unknown input, we did the analysis for the constant case and with two non-zero derivatives. In both situations we find that the model is FISPO with both of the algorithms. FISPO spent 0,5 seconds running for the constant case and 0,83 for time-varying one, while *ProbObsTest* required 5,33 and 9,48, respectively.

Also, to check the behaviour with more than one unknown input, we have adapted the model taking the known input as unknown. For the constant case we found that x_2 is non observable and F_1 and F_2 are not reconstructible. The solution obtained matches the one achieved with the previous implementation. FISPO took 0,5 seconds running while the *ProbObsTest* took 5,33. With two non-zero derivatives we found that $\dot{x_2}$ and x_2 are unobservable, and F_1, $\dot{F_1}$, F_2 and $\dot{F_2}$ are not reconstructible. The times are now 18,9 for STRIKE-GOLDD, and 20,79 for the new code. *ObservabilityTest* cannot be applied to this model since it has unknown inputs.

Pharmacokinetics model (PK): This example is taken from [14]. It is a model of the behaviour of certain orally administered drugs. It consists of:

- 4 states, $x = (x_1; x_2; x_3; x_4)$,
- 10 parameters, $\theta = (k_1; k_2; k_3; k_4; k_5; k_6; k_7; s_2; s_3)$,
- 1 input, u, that will be considered known and unknown,
- 2 outputs, $g(x, \theta, w) = (s_2 \cdot x_2; s_3 \cdot x_3)$,

and it is governed by the following state equations:

$$f(x, \theta, u, w) = \begin{pmatrix} u_1 - (k_1 + k_2) \cdot x_1 \\ k_1 \cdot x_1 - (k_3 + k_6 + k_7) \cdot x_2 + k_5 \cdot x_4 \\ k_2 \cdot x_1 + k_3 \cdot x_2 - k_4 \cdot x_3 \\ k_6 \cdot x_2 - k_5 \cdot x_4 \end{pmatrix}.$$

For the known input case we obtain that x_2, x_3 and x_4 are unobservable and k_1, k_2, k_3, k_7, s_2 and s_3 are unidentifiable. The execution times are 1,95 seconds for STRIKE-GOLDD, 5,81 seconds for *ProbObsTest* and 0,141 seconds for *ObservabilityTest*. When u is an unknown constant input only k_4, k_5 and k_6 are identifiable, all the states are unobservable, and the input is not reconstructible. FISPO takes 6,54 seconds and *ProbObsTest* 7,3. With 3 non-zero derivatives of the input we get the same result; FISPO takes 14,75 seconds and *ProbObsTest* 16,8.

SIRS model: In [2] the transmission of respiratory syncytial virus (RSV) is modeled. We work with the model that takes into account the seasonal nature of transmission through an oscillating contact rate. The population is divided into susceptible (S), infected and infectious (I), and recovered (R) individuals. The model consists of:

- 5 states, $x = (S; I; R; x_1; x_2)$,
- 10 parameters, $\theta = (\nu; b_1; b_0; M; \mu; g)$,
- 0 inputs,
- 2 outputs, $g(x, \theta, w) = (I; R)$,

and it is governed by the following state equations:

$$f(x,\theta,u,w) = \begin{pmatrix} \mu - S\cdot\mu - b_0\cdot(1+b_1\cdot x_1)\cdot S\cdot I + g\cdot R \\ b_0\cdot(1+b_1\cdot x_1)\cdot S\cdot I - (\nu+\mu)\cdot I \\ \nu\cdot I - (\mu+g)\cdot R \\ -M\cdot x_2 \\ M\cdot x_1 \end{pmatrix}.$$

The three implementations find b_1 unidentifiable and x_1 and x_2 unobservable. STRIKE-GOLDD took 70,56 seconds, *ProbObsTest* 7,55 and *ObservabilityTest* 0,125.

Markov model: These models are used to describe how ion channels regulate the flow of ions across cell membranes. We are using the model from [22], where a reduction of a cyclic Markov model is considered. The model consists of:

- 2 states, $x = (x_1; x_2)$,
- 10 parameters, $\theta = (a_{12}; a_{21}; b_{12}; b_{21}; a_{23}; a_{32}; b_{23}; b_{32}; a_{13}; b_{13})$,
- 1 known input, u_1,
- 1 output, $g(x,\theta,w) = x_1$,

and it is governed by the following state equations:

$$f(x,\theta,u,w) = \begin{pmatrix} x_2\cdot e^{a_{21}+b_{21}\cdot u_1} - x_1\cdot(e^{a_{12}+b_{12}\cdot u_1} + e^{a_{13}+b_{13}\cdot u_1})\ldots \\ \ldots - e^{a_{13}-a_{12}+a_{21}-a_{23}+a_{32}+u_1\cdot(b_{13}-b_{12}+b_{21}-b_{23}+b_{32})}\cdot(x_1+x_2-1) \\ x_1\cdot e^{a_{12}+b_{12}\cdot u_1} - e^{a_{32}+b_{32}\cdot u_1}\cdot(x_1+x_2-1) - x_2\cdot(e^{a_{21}+b_{21}\cdot u_1} + e^{a_{23}+b_{23}\cdot u_1}) \end{pmatrix}.$$

This example highlights the different behaviour of the algorithms in regard to the number of derivatives for the known inputs.

For the constant known input case, the analysis with FISPO takes 624,11 seconds. *ProbObsTest* gets the same results in only 18,65 seconds.

With an infinite number of derivatives, FISPO is not able to do the analysis due to an out of memory error. In contrast, *ProbObsTest* seems unaffected by this change, and concludes the analysis within 18,49 seconds. In [22] the time-varying input the study is accomplished performing 4 experiments with different constant inputs observing identifiability as the probabilistic method. This example can be analysed with *ProbObsTest* using the automatic reformulation for non rational systems.

NF-κB model: NF-κB stands for "nuclear factor kappa-light-chain-enhancer of activated B cells". We use as reference the analysis done with STRIKE-GOLDD in [21] based in [9]. The model consists of:

- 15 states, $x = (x_1; x_2; x_3; x_4; x_5; x_6; x_7; x_8; x_9; x_{10}; x_{11}; x_{12}; x_{13}; x_{14}; x_{15})$,
- 29 parameters, $\theta = (t_1; t_2; c_{3a}; c_{4a}; c_5; k_1; k_2; k_3; k_{prod}; k_{deg}; i_1; e_{2a}; i_{1a}; \ldots$ $a_1; a_2; a_3; c_{1a}; c_{2a}; c_{5a}; c_{6a}; c_1; c_2; c_3; c_4; k_v; e_{1a}; c_{1c}; c_{2c}; c3c)$,
- 1 known input, u_1,
- 6 outputs: $g(x,\theta,u,w) = \begin{pmatrix} x_7 \\ x_{10}+x_{13} \\ x_9 \\ x_1+x_2+x_3 \\ x_2 \\ x_{12} \end{pmatrix}$,

and it is governed by the following state equations:

$$f(x,\theta,u,w) = \begin{pmatrix} k_{prod} - k_{deg}\cdot x_1 - k_1\cdot x_1\cdot u_1 \\ -k_3\cdot x_2 - k_{deg}\cdot x_2 - a_2\cdot x_2\cdot x_{10} + t_1\cdot x_4 - a_3\cdot x_2\cdot x_{13}\dots \\ \dots + t_2\cdot x_5 + (k_1\cdot x_1 - k_2\cdot x_2\cdot x_8)\cdot u_1 \\ k_3\cdot x_2 - k_{deg}\cdot x_3 + k_2\cdot x_2\cdot x_8\cdot u_1 \\ a_2\cdot x_2\cdot x_{10} - t_1\cdot x_4 \\ a_3\cdot x_2\cdot x_{13} - t_2\cdot x_5 \\ c_{6a}\cdot x_{13} - a_1\cdot x_6\cdot x_{10} + t_2\cdot x_5 - i_1\cdot x_6 \\ i_1\cdot k_v\cdot x_6 - a_1\cdot x_{11}\cdot x_7 \\ c_4\cdot x_9 - c_5\cdot x_8 \\ c_2 + c_1\cdot x_7 - c_3\cdot x_9 \\ -a_2\cdot x_2\cdot x_{10} - a_1\cdot x_{10}\cdot x_6 + c_{4a}\cdot x_{12}\dots \\ \dots - c_{5a}\cdot x_{10} - i_{1a}\cdot x_{10} + e_{1a}\cdot x_{11} \\ -a_1\cdot x_{11}\cdot x_7 + i_{1a}\cdot k_v\cdot x_{10} - e_{1a}\cdot k_v\cdot x_{11} \\ c_{2a} + c_{1a}\cdot x_7 - c_{3a}\cdot x_{12} \\ a_1\cdot x_{10}\cdot x_6 - c_{6a}\cdot x_{13} - a_3\cdot x_2\cdot x_{13} + e_{2a}\cdot x_{14} \\ a_1\cdot x_{11}\cdot x_7 - e_{2a}\cdot k_v\cdot x_{14} \\ c_{2c} + c_{1c}\cdot x_7 - c_{3c}\cdot x_{15} \end{pmatrix}.$$

For this model the execution time with FISPO is 2866,55 seconds while for *ProbObsTest* is 279,51 and for *ObservabilityTest* 8,422 seconds. We found that states x_8 and x_{15} are unobservable and k_2, c_4, c_{1c}, c_{2c} and c_{3c} are unidentifiable. We also did the analysis with parameters a_1, a_2, a_3, c_{1a}, c_{2a}, c_{5a}, c_{6a}, c_1, c_2, c_3, c_4, k_v, e_{1a}, c_{1c}, c_{2c} and c_{3c} fixed to some specific values. In this case we have that x_{15} is unobservable. For the second model the times are 844,46, 291,21 and 3,14, respectively.

βIG model: This example is one of the four case studies presented in [7], representing a physiological circuit that models possible regulatory mechanisms of glucose homeostasis. It was also included in [20]. It consists of:

- 3 states, $x = (G;\beta;I)$,
- 5 parameters, $\theta = (p;s_i;\gamma;c;\alpha)$,
- 1 input, called u that will be considered both as known and unknown,
- 1 output, $g(x,\theta,w) = G$,

and it is governed by the following state equations:

$$f(x,\theta,u,w) = \begin{pmatrix} u - (c + s_i\cdot I)\cdot G \\ \beta\cdot \dfrac{\frac{0{,}021}{24\cdot 60}}{1+(\frac{8{,}4}{G})^{1{,}7}} - \dfrac{\frac{0{,}025}{24\cdot 60}}{1+(\frac{G}{4{,}8})^{8{,}5}} \\ \dfrac{p\cdot\beta\cdot G^2}{\alpha^2+G^2} - \gamma\cdot I \end{pmatrix}.$$

With this model we are going to analyse different cases. For all of them the results with the two algorithms match. With the input as known, FISPO takes 984,89 seconds while *ProbObsTest* only 12,65. For constant unknown input these times were 14140,08 and 21,85 seconds, respectively. With three non-zero derivatives of the unknown input, FISPO yields an out of memory error while *ProbObsTest* takes 38,95 seconds. Here we have the best exhibit of the improvements that the new implementation can achieve. For this relatively small but complex system, even for the known input case we see that a great computational acceleration is obtained. Moreover, with the unknown input with three non-zero derivatives we reach a point where FISPO cannot analyse the model while the new algorithm can. Finally, in order to analyse this model with *ObservabilityTest* we need to approximate the non-integer exponents in the equations with integer values. Otherwise, the code issues an error but it gives no clue about its cause or possible fix. After fixing it, the analysis took 0,016 seconds.

JAKSTAT model: This model is introduced in [1] and consists of:

- 25 states, $x = (EpoRJAK2; EpoRpJAK2; p1EpoRpJAK2; p2EpoRpJAK2; p12EpoRpJAK2; EpoRJAK2_CIS; SHP1; SHP1Act; STAT5; pSTAT5; npSTAT5; CISnRNA1; CISnRNA2; CISnRNA3; CISnRNA4; CISnRNA5; CISRNA; CIS; SOCS3nRNA1; SOCS3nRNA2; SOCS3nRNA3 ; SOCS3nRNA4; SOCS3nRNA5; SOCS3RNA; SOCS3)$,
- 27 parameters, $\theta = (CISEqc; CISEqcOE; CISInh; CISRNADelay; CISRNATurn; CISTurn; EpoRActJAK2; EpoRCISInh; EpoRCISRemove; JAK2ActEpo; JAK2EpoRDeaSHP1; SHP1ActEpoR; SHP1Dea; SHP1ProOE; SOCS3Eqc; SOCS3EqcOE; SOCS3Inh; SOCS3RNADelay; SOCS3RNATurn; SOCS3Turn; STAT5ActEpoR; STAT5ActJAK2; STAT5Exp; STAT5Imp; init_EpoRJAK2; init_SHP1; init_STAT5)$,
- 5 known inputs, $u = (ActD; CISoe; SOCS3oe; SHP1oe; Epo)$,
- 0 unknown inputs,
- 14 outputs, with equations:

$$g(x,\theta,w) = \begin{pmatrix} \frac{2\cdot(EpoRpJAK2 + p12EpoRpJAK2 + p1EpoRpJAK2 + p2EpoRpJAK2)}{init_EpoRJAK2} \\ \frac{16\cdot(p12EpoRpJAK2 + p1EpoRpJAK2 + p2EpoRpJAK2)}{init_EpoRJAK2} \\ \frac{SOCS3}{SOCS3Eqc} \\ \frac{STAT5 + pSTAT5}{init_STAT5} \\ \frac{pSTAT5}{init_STAT5} \\ STAT5 \\ SHP1 + SHP1Act \\ CIS \\ SOCS3 \\ \frac{100\cdot pSTAT5}{STAT5 + pSTAT5} \\ SOCS3RNA \\ CISRNA \\ \frac{(SHP1 + SHP1Act)\cdot(SHP1oe\cdot SHP1ProOE + 1)}{init_SHP1} \\ \frac{CIS}{CISEqc} \end{pmatrix}.$$

For state equations we will refer to [18]. To analyse this model we consider the last parameter, which is the initial condition of one of the measured states, as known. FISPO yields an out of memory error; to avoid it, the analysis has to be done decomposing the model. In contrast, *ProbObsTest* takes 1914,79 seconds and *ObservabilityTest* just a few seconds.

CHO model: This is a metabolic model of Chinese Hamster Ovary (CHO) cells, which are used for protein production in fermentation processes [23]. The model consists of:

- 32 states, $x = (x_1; \ldots; x_{32})$,
- 117 parameters, $\theta = (\theta_1; \ldots; \theta_{117})$,
- 0 inputs,
- 13 outputs, $g(x,\theta,w) = (x_5; x_4; x_3; x_2; x_1; x_{29}; x_{27}; x_{21}; x_{15}; x_{13}; x_{30}; x_{32}; x_{11})$.

The state equations were modelled with in lin-log kinetics. The resulting expressions are non-rational, due to the presence of logarithms, so *ObservabilityTest* cannot be applied unless the model is reformulated. STRIKE-GOLDD 4.0 checks for this possibility and reformulates the model automatically.

In [21], the analysis of the model with STRIKE-GOLDD (i.e. FISPO) was only partially accomplished. When trying to analyse the whole model FISPO gives an out-of-memory error. After decomposing it in smaller submodels, four parameters were found to be unidentifiable, 95 were classified as identifiable, and the identifiability of the remaining 18 could not be determined. (After fixing six of them, the model was found to be FISPO.)

In contrast, *ProbObsTest* can analyse the whole model, obtaining conclusive results for all parameters without the need for decomposition. However, it should be noted that it took 12 days to conclude the analysis, which reached the 99% of the computer memory.

3.2 Synergies created by using *ProbObsTest* within STRIKE-GOLDD

3.2.1 Application to models with unknown polynomial inputs of high-degree

To show how *ProbObsTest* outperforms FISPO when working with high-degree unknown inputs, we have increased the degree of the unknown polynomial input in our analyses of model C2M for the case with known b. Figure 2 shows a table and a graph of the execution times for the two algorithms for different degrees of the unknown polynomial input.

	Algorithm	
Degree	FISPO	ProbObsTest
0	0,67	1,4
3	2,14	4,19
5	8,83	7,33
10	526,54	17,23
15	43336,36	44,54

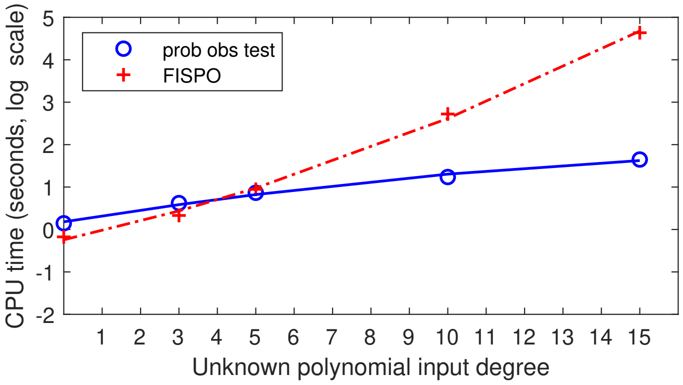

Figure 2: Comparison of times for the analysis of unknown polynomial inputs of different degrees for the C2M model with an unknown input and parameter b known.

We analysed this example in detail because of its comparatively low computation times, which allow us to increase the degree significantly. As can be seen, *ProbObsTest* effectively is much faster than FISPO for unknown polynomial inputs of high degree, and the difference is larger for larger degrees. We observe a similar trend in other models, such as 2DOF with 2 unknown inputs (examples 8 and 13 of Figure 1.B of the main text) and βIG (examples 16 and 20).

3.2.2 Application to nonrational models

The automatic conversion of nonrational to rational models enables their analysis with *ProbObsTest*. We demonstrate the importance of this new feature with two different models.

The analysis of the CHO model, which is very large (117 parameters) and contains logarithmic functions, cannot be fully completed with the FISPO algorithm. In contrast, *ProbObsTest* completes a full analysis (even though it took around 12 days in our computer).

In the Markov model the complication is not given by its size (it is not a large model, with ten parameters) but by the complexity of its equations. FISPO can analyse the constant input case, but not the time-varying input case. Thanks to the automatic reformulation of non rational models and the new method, the toolbox is able to do both analyses in just around 18 seconds each.

Thus, the capability to transform some nonrational models into rational models can yield exceptional gains. However, this feature has to be used carefully, since as was previously mentioned in some cases the identifiability properties of the model can be modified by the transformation.

3.2.3 Application within AutoRepar

AutoRepar [12] is a method available in STRIKE-GOLDD that seeks for Lie symmetries in the model equations and removes them in order to yield a fully observable model. In previous versions of STRIKE-GOLDD the necessary SIO analysis was performed with FISPO. Since AutoRepar

requires the models to be rational, in STRIKE-GOLDD 4.0 the analysis is performed with *ProbObsTest*. This modification yields considerable accelerations, as demonstrated by the following two case studies:

With the βIG model, AutoRepar takes 36 seconds when using *ProbObsTest* in comparison with the 1663 seconds required by FISPO. This improvement is entirely attributable to the identifiability analysis. The outcome can be found in [12].

For the HIV model with 5 states, running AutoRepar with FISPO takes 16078 seconds, while with *ProbObsTest* it takes only 413 seconds. In the following we discuss the results. Four parameters are found to be unidentifiable (β, λ, c, k), and three states unobservable (xx, y, v). The analysis tells us that two transformations are needed, and it finds two generators with two and three different parameters to be removed, respectively. We choose to remove parameter k using the first generator. The following changes are made:

$$\begin{aligned} &v \to v/k \\ &\beta \to \beta \cdot k \\ &k \to 1 \end{aligned}$$

The equations of the reformulated model are:

$$f(x,\theta,u,w) = \begin{pmatrix} \lambda - (d \cdot xx) - (\beta \cdot xx \cdot v) \\ (\beta \cdot xx \cdot v) - (a \cdot y) \\ y - (uu \cdot v) \\ (c \cdot z \cdot y \cdot ww) - (c \cdot q \cdot y \cdot ww) - (b \cdot ww) \\ (c \cdot q \cdot y \cdot ww) - (hh \cdot z) \end{pmatrix}$$

$$g(x,\theta,w) = (ww; z)$$

For the new model, three parameters are found to be unidentifiable (β, λ, c), and three states unobservable (xx, y, v). The method finds one generator with three different parameters to be removed. We choose to remove parameter β. The following changes are made:

$$\begin{aligned} &xx \to \beta \cdot xx \\ &y \to \beta \cdot y \\ &v \to \beta \cdot v \\ &\beta \to 1 \\ &\lambda \to \beta \cdot \lambda \\ &c \to c/\beta \end{aligned}$$

The resulting model is FISPO, and its equations are:

$$f(x,\theta,u,w) = \begin{pmatrix} \lambda - (d \cdot xx) - (xx \cdot v) \\ (xx \cdot v) - (a \cdot y) \\ y - (uu \cdot v) \\ (c \cdot z \cdot y \cdot ww) - (c \cdot q \cdot y \cdot ww) - (b \cdot ww) \\ (c \cdot q \cdot y \cdot ww) - (hh \cdot z) \end{pmatrix}$$

$$g(x,\theta,w) = (ww; z)$$

References

[1] J. Bachmann, A. Raue, M. Schilling, M. E. Böhm, C. Kreutz, D. Kaschek, H. Busch, N. Gretz, W. D. Lehmann, J. Timmer, et al. Division of labor by dual feedback regulators controls jak2/stat5 signaling over broad ligand range. *Molecular systems biology*, 7(1):516, 2011.

[2] M. A. Capistrán, M. A. Moreles, and B. Lara. Parameter estimation of some epidemic models. the case of recurrent epidemics caused by respiratory syncytial virus. *Bulletin of mathematical biology*, 71(8):1890–1901, 2009.

[3] M. N. Chatzis, E. N. Chatzi, and A. W. Smyth. On the observability and identifiability of nonlinear structural and mechanical systems. *Structural Control and Health Monitoring*, 22(3):574–593, 2015.

[4] M. N. Chatzis, E. N. Chatzi, and A. W. Smyth. On the observability and identifiability of nonlinear structural and mechanical systems. *Structural Control and Health Monitoring*, 22(3):574–593, 2015.

[5] S. Diop and M. Fliess. Nonlinear observability, identifiability, and persistent trajectories. In *[1991] Proceedings of the 30th IEEE Conference on Decision and Control*, pages 714–719. IEEE, 1991.

[6] H. Hong, A. Ovchinnikov, G. Pogudin, and C. Yap. SIAN: software for structural identifiability analysis of ODE models. *Bioinformatics*, 35(16):2873–2874, 01 2019.

[7] O. Karin, A. Swisa, B. Glaser, Y. Dor, and U. Alon. Dynamical compensation in physiological circuits. *Molecular systems biology*, 12(11):886, 2016.

[8] J. Karlsson, M. Anguelova, and M. Jirstrand. An efficient method for structural identifiability analysis of large dynamic systems. *IFAC proceedings volumes*, 45(16):941–946, 2012.

[9] T. Lipniacki, P. Paszek, A. R. Brasier, B. Luxon, and M. Kimmel. Mathematical model of nf-κb regulatory module. *Journal of theoretical biology*, 228(2):195–215, 2004.

[10] K. Maes, M. Chatzis, and G. Lombaert. Observability of nonlinear systems with unmeasured inputs. *Mech. Syst. Signal Process.*, 130:378–394, 2019.

[11] G. Margaria, E. Riccomagno, and L. J. White. Structural identifiability analysis of some highly structured families of statespace models using differential algebra. *Journal of mathematical biology*, 49(5):433–454, 2004.

[12] G. Massonis, J. R. Banga, and A. F. Villaverde. Repairing dynamic models: a method to obtain identifiable and observable reparameterizations with mechanistic insights. *arXiv preprint arXiv:2012.09826*, 2020.

[13] H. Miao, X. Xia, A. S. Perelson, and H. Wu. On identifiability of nonlinear ode models and applications in viral dynamics. *SIAM review*, 53(1):3–39, 2011.

[14] A. Raksanyi. *Utilisation du calcul formel pour l'étude des systèmes d'équations polynomiales (applications en modélisation)*. PhD thesis, Paris 9, 1986.

[15] A. Sedoglavic. A probabilistic algorithm to test local algebraic observability in polynomial time. *J. Symbol. Comput.*, 33(5):735–755, 2002.

[16] X. Shi and M. Chatzis. An efficient algorithm to test the observability of rational nonlinear systems with unmeasured inputs. *Mech. Syst. Signal Process.*, 165:108345, 2022.

[17] E. Tunali and T.-J. Tarn. New results for identifiability of nonlinear systems. *IEEE Transactions on Automatic Control*, 32(2):146–154, 1987.

[18] A. Villaverde and J. R. Banga. Análisis de observabilidad e identificabilidad estructural de modelos no lineales: aplicación a la vía de señalización jak/stat. In *XL Jornadas de Automática*, pages 631–638. Universidade da Coruña, Servizo de Publicacións, 2019.

[19] A. F. Villaverde. Observability and structural identifiability of nonlinear biological systems. *Complexity*, 2019, 2019.

[20] A. F. Villaverde and J. R. Banga. Dynamical compensation and structural identifiability of biological models: Analysis, implications, and reconciliation. *PLoS computational biology*, 13(11), 2017.

[21] A. F. Villaverde, A. Barreiro, and A. Papachristodoulou. Structural identifiability of dynamic systems biology models. *PLoS Comput. Biol.*, 12(10):e1005153, 2016.

[22] A. F. Villaverde, N. D. Evans, M. J. Chappell, and J. R. Banga. Input-dependent structural identifiability of nonlinear systems. *IEEE Control Systems Letters*, 3(2):272–277, 2018.

[23] A. F. Villaverde, D. Henriques, K. Smallbone, S. Bongard, J. Schmid, D. Cicin-Sain, A. Crombach, J. Saez-Rodriguez, K. Mauch, E. Balsa-Canto, et al. Biopredyn-bench: a suite of benchmark problems for dynamic modelling in systems biology. *BMC systems biology*, 9(1):1–15, 2015.

[24] A. F. Villaverde, N. Tsiantis, and J. R. Banga. Full observability and estimation of unknown inputs, states and parameters of nonlinear biological models. *J. Roy. Soc. Interface*, 16(156):20190043, 2019.

[25] D. Wodarz and M. A. Nowak. Mathematical models of hiv pathogenesis and treatment. *BioEssays*, 24(12):1178–1187, 2002.
